# Microtubule integrity regulates budding yeast RAM pathway gene expression

**DOI:** 10.3389/fcell.2022.989820

**Published:** 2022-09-12

**Authors:** Cameron Howard Lee, Sue Biggins

**Affiliations:** Division of Basic Sciences, Fred Hutchinson Cancer Center, Howard Hughes Medical Institute, Seattle, WA, United States

**Keywords:** microtubule, ribosome profiling, RAM pathway, translation, transcription, mitosis, Cbk1

## Abstract

During mitosis, cells must spatiotemporally regulate gene expression programs to ensure accurate cellular division. Failures to properly regulate mitotic progression result in aneuploidy, a hallmark of cancer. Entry and exit from mitosis is largely controlled by waves of cyclin-dependent kinase (CDK) activity coupled to targeted protein degradation. The correct timing of CDK-based mitotic regulation is coordinated with the structure and function of microtubules. To determine whether mitotic gene expression is also regulated by the integrity of microtubules, we performed ribosome profiling and mRNA-sequencing in the presence and absence of microtubules in the budding yeast *Saccharomyces cerevisiae*. We discovered a coordinated translational and transcriptional repression of genes involved in cell wall biology processes when microtubules are disrupted. The genes targeted for repression in the absence of microtubules are enriched for downstream targets of a feed-forward pathway that controls cytokinesis and septum degradation and is regulated by the Cbk1 kinase, the Regulation of Ace2 Morphogenesis (RAM) pathway. We demonstrate that microtubule disruption leads to aberrant subcellular localization of Cbk1 in a manner that partially depends on the spindle position checkpoint. Furthermore, constitutive activation of the RAM pathway in the absence of microtubules leads to growth defects. Taken together, these results uncover a previously unknown link between microtubule function and the proper execution of mitotic gene expression programs to ensure that cell division does not occur prematurely.

## Introduction

Mitosis, the process by which genetic information is passed on from one cell to the next, is a highly coordinated and regulated event (McIntosh, 2016). Progression through mitosis is carefully controlled because errors result in aneuploidy, a hallmark of cancer and other diseases (Hanahan and Weinberg, 2011; Holland and Cleveland, 2012). This process is driven by increasing cyclin-dependent kinase (CDK) activity that needs to be reversed to allow mitotic exit. CDK activity is regulated by the Anaphase Promoting Complex (APC), an E3 ubiquitin ligase that targets cyclins for degradation and activates phosphatases such as Cdc14 to reverse CDK phosphorylation (Peters, 2006; Curtis and Bolanos-Garcia, 2019; Lara-Gonzalez et al., 2021). While the roles of kinase and opposing phosphatase activities in mitotic progression are well understood, the contribution of other regulatory mechanisms such as translational control is less well studied. Although translation is globally repressed during mitosis (Prescott and Bender, 1962; Fan and Penman, 1970; Stumpf et al., 2013; Tanenbaum et al., 2015), ribosomes and newly synthesized proteins have been observed to colocalize with the mitotic apparatus, suggesting that specific transcripts are locally translated during mitosis (Roth and Daniels, 1962; Gross and Cousineau, 1963; Stafford et al., 1964; Mangan et al., 1965). In addition, microtubules are important for localized translation during development in metazoans (Das et al., 2021), suggesting a functional link between microtubules and translational regulation. However, it is not known whether there is microtubule-dependent regulation of translation during mitosis. We therefore set out to analyze this in budding yeast, an organism that has been widely used to study cell cycle progression and translation due to its simple genome and ease of genetic manipulation (Hartwell et al., 1973; Hinnebusch, 2005; Altmann and Linder, 2010).

Mitosis in budding yeast is regulated by coordinated signaling pathways that ensure the cell cycle does not progress if microtubule-based processes are disrupted. The spindle assembly checkpoint (SAC) monitors attachments of the mitotic spindle to kinetochores, the large macromolecular complexes that assemble on centromeric DNA (Musacchio and Desai, 2017; Lara-Gonzalez et al., 2021). The SAC signaling cascade prevents the onset of anaphase when there are spindle assembly defects by inhibiting Cdc20, an APC activator. To ensure that chromosomes are accurately delivered to the daughter cell, the mitotic exit network (MEN) coordinates spindle position and orientation with release of the Cdc14 phosphatase from the nucleolus (Hotz and Barral, 2014). If the spindle is misaligned, the spindle position checkpoint inhibits the MEN *via* a GTPase activating complex called Bub2/Bfa1 (Bloecher et al., 2000; Pereira et al., 2000; Wang et al., 2000). After chromosome segregation, the cell wall must be remodeled to promote cytokinesis. These events are controlled by the **r**egulation of **A**ce2 transcription factor and polarized morphogenesis (RAM) pathway (Racki et al., 2000; Bidlingmaier et al., 2001; Colman-Lerner et al., 2001; Weiss et al., 2002). RAM signaling is activated by the Ndr/LATS kinase Cbk1 that promotes the transcription of genes involved in cell wall function by phosphorylating the Ace2 transcription factor (Colman-Lerner et al., 2001; Nelson et al., 2003; Sbia et al., 2008). In addition, Cbk1 also promotes the translation of these genes by inhibiting the Ssd1 translational inhibitor (Jansen et al., 2009; Kurischko et al., 2011). To ensure that cell wall remodeling occurs after chromosome segregation, the RAM pathway is inhibited during mitosis by high Cdk1 activity and is then activated during mitotic exit by the Cdc14 phosphatase (Mancini Lombardi et al., 2013).

We set out to test whether there is microtubule-dependent translational regulation during budding yeast mitosis since other mitotic signaling pathways are regulated by the integrity of the microtubule cytoskeleton. Towards this end, we performed ribosome profiling in mitotically arrested budding yeast cells treated with the microtubule destabilizing drug nocodazole. We identified the translational downregulation of a functionally related set of mRNAs upon microtubule disruption and find that many of these mRNAs are also transcriptionally repressed. A subset of the mRNAs are targets of the RAM signaling pathway. Consistent with this, we found that microtubule disruption leads to altered Cbk1 localization in a manner that is partially dependent on the mitotic exit network. Forced activation of the RAM pathway in the presence of microtubule disruption impaired cell growth. Taken together, our data suggest that there is a coordinated program that regulates gene expression when microtubules are disrupted to ensure the accurate coordination of mitotic events.

## Materials and methods

### Yeast methods and drug treatments

Yeast were standardly cultured in YEP + 1% adenine + 2% glucose at 23°C. Yeast strains were constructed using standard genetic techniques. The auxin inducible degron (AID) system was used as previously described (Nishimura et al., 2009). All strains can be found in Supplementary Table S1. To degrade AID tagged proteins, 500 μM IAA (indole-3-acetic acid; Sigma Aldrich #I3750-5G-A) was added to media. Nocodazole (10 μg/ml in DMSO; Sigma Aldrich #M1404-50MG) was used to depolymerize microtubules. Cell cycle arrests (from auxin or nocodazole) were confirmed *via* microscopy for each experiment. As a control, DMSO was used at equal volume to amount of nocodazole added. All drug treatments were performed for 2.5 h.

### Microscopy

Yeast were cultured in YC +1% Adenine +2% glucose + 1× CSM (Sunrise Science Products #1001-100). Yeast were imaged live on agarose pads (1.3% agarose, 4% glucose) containing 2× concentration of drugs as needed (e.g., 20 μg/ml nocodazole; 1 M auxin). Cells were imaged using a Deltavision Ultra deconvolution high-resolution microscope equipped with a 60× or 100×/1.40 UPlanSApo oil-immersion objective (Olympus). Images were captured using a 16-bit sCMOS detector camara. Cells were imaged with z-stacks through entire cells using 0.2 μm steps. Images were deconvolved using standard settings. Images were processed in FIJI for background subtraction and uniform brightness and contrast adjustments applied to entire images.

### RNA extractions and quantitative real-time PCR assay

RNA was purified from 5 ml of culture harvested at OD_600_ ∼0.8 using a Direct-zol RNA miniprep kit with on-column DNase treatment (Zymo #R2050). 1 μg of RNA was then reverse transcribed using an oligo dT primer (Thermo Fisher Scientific #FERSO132) and Protoscript II (200 μ/μl; New England Biolabs #M0368L) in a 20 μl reaction for 30 min at 50°C. Samples were diluted to 120 μl with dH2O prior to qRT-PCR that was performed on an ABI QuantStudio5 instrument in the Fred Hutchinson Cancer Research Center Genomics Core facility. Genes were amplified using primers listed in Supplementary Table S2. Primers were validated to amplify a single amplicon *via* melt curve analysis, and quantification was performed using standard curves for each primer set.

### Motif discovery and ChIP-seq analysis

Promoters of differentially expressed genes (defined as −500 to +1) were downloaded from the *S. cerevisiae* promoter database (http://rulai.cshl.edu/SCPD/) and were used as input into HOMER v4.11 findMotifs.pl (Heinz et al., 2010). Genes known to be a part of the Environmental Stress Response (Gasch et al., 2000) were excluded from the analysis.

ChIP-seq data from asynchronous cells for Swi5-TAP, Ace2-TAP, and Swi4-TAP (sample IDs: 18061, 16021 and 12000 respectively) was downloaded from the Yeast Epigenome Project (Rossi et al., 2021). ChIP-seq heatmaps were generated using deeptools (version 3.3.0) (Ramirez et al., 2016) commands “computeMatrix reference-point -b 1000 -a 200—missingDataAsZero” and “plotHeatmap—zMin −1 –zMax 3—sortUsing max—sortRegions descend”.

### Ribosome profiling

200 ml (YEP + 1% Adenine + 2% glucose) of an asynchronous culture of SBY14004 was grown at 23°C to OD_600_ of ∼0.2. Cultures were then treated with 500 μM Indole-3-Acetic Acid and 10 μg/ml nocodazole for 2.5 h. Arrests were confirmed *via* microscopy. Cultures were then harvested *via* vacuum filtration onto nitrocellulose membranes and scraped immediately into liquid nitrogen.

Pellets were lysed at 4 °C *via* vortexing with glass microbeads in 1x lysis buffer (1% Triton-X 100; 0.1% sodium deoxycholate; 20 mM Tris pH 7.5; 150 mM KCl; 15 mM MgCl_2_; 100 μg/ml cycloheximide; 2 mM PMSF; 1X LPC). Lysates were clarified for 30 min at 16000 rpm at 4°C. Clarified lysates were then snap-frozen in liquid nitrogen until ultra-centrifugation.

10%–50% sucrose gradients in 1× polysome buffer (20 mM Tris pH 7.5; 150 mM KCl; 15 mM MgCl_2_; 100 μg/ml cycloheximide; 2 mM PMSF; 1X LPC) were made in ultracentrifuge tubes (Seton #NC9863486) with a BioComp Gradient Master and used immediately. 400 μg of RNA was then layered over the gradients and loaded onto an SW41 rotor. Samples were ultracentrifuged at 35000 rpm for 2.5 h at 4°C. Following ultracentrifugation, samples were fractionated at 0.3 mm/s with UV absorbance monitoring at 254 nm (EconoUV monitor).

To generate ribosome profiling sequencing libraries, prior to fractionation, 400 μg of RNA was first treated with 2 μl RNase I (10 U/μL; Lucigen #N6901K) for 45 min at room temperature. After digestion, SUPERase RNA inhibitor was added (200 U; Life Technologies #AM2694). Monosome fractions were then collected as described above. RNA was extracted from monosome fraction with TRIzol (Thermo Fisher Scientific #15-596-018) following manufacturer’s instructions. Ribosome profiling and input mRNA-seq libraries were generated from biological triplicates according to a previously published protocol (McGlincy and Ingolia, 2017). Briefly, 1 μg of RNA was size selected on a precast 15% polyacrylamide TBE-Urea gel (Thermo Fisher Scientific #EC68855BOX) using markers NI801 and NI800 for 26 and 34 bases, respectively. Size selected RNAs were purified from the gel overnight in RNA extraction buffer (300 mM Sodium acetate, pH 5.5; 1 mM EDTA; 0.25% SDS v/v) with rotation at room temperature. The following day, RNA was dephosphorylated with T4 Polynucleotide Kinase (New England Biolabs #M0201L) for 1 h at 37°C. Dephosphorylated RNA was then ligated to pre-adenylated, barcoded linker oligos (NI810, NI811, NI812 for DMSO replicates and NI813, NI814, NI815 for nocodazole replicates) for 3 h at 22°C using T4 Rnl2 (tr) K227Q (New England Biolabs #M0351L). Unligated linkers were then depleted at 30°C for 45 min after addition of 0.5 μl of yeast 5′-deadenylase (10 U/μl; New England Biolabs #M0331S) and 0.5 μl RecJ exonuclease (10 U/μl; Lucigen #RJ411250) directly to ligation reaction. Samples were then pooled and purified over a Zymo Oligo Clean & Concentrator column (Genesee Scientific #11-380) according to manufacturer’s instructions. rRNA was subsequently depleted as previously described (Thompson et al., 2020) using biotinylated oligos oSB6755-oSB6769. rRNA-depleted samples were then reverse transcribed in 20 uL reactions with primer NI802 and Protoscript II (200 U/μl) for 30 min at 50°C. RNA was hydrolyzed by addition of 2.2 μl 1 M sodium hydroxide and incubated at 70°C for 20 min cDNA was purified over a Zymo DNA Clean and concentrator column (Genesee Scientific #11-302). RT products were then size selected on a 15% TBE-UREA gel (Thermo Fisher Scientific #EC68855BOX) and purified overnight in DNA extraction buffer (300 mM NaCl, 10 mM Tris pH 8, 1 mM EDTA) with rotation at room temperature. The following day, DNA was circularized using CircLigase II (100 U/μl; Lucigen #CL9021K) for 1 h at 60°C followed by heat inactivation for 10 min at 80°C. The concentration of circularized libraries was quantified using qRT-PCR and oligos NI827/NI828, with a dilution series of NI803 as a standard curve. Libraries were amplified forward primer NI798 and reverse index primers NI799 and NI822 for 11 PCR cycles. Purified DNA libraries were then sequenced on a single Illumina NextSeq run.

### Sequencing analysis

Adapters were trimmed with cutadapt v2.9 (Martin, 2011). Subsequently, DMSO treated versus nocodazole treated samples were demultiplexed based on barcoded oligos NI810-NI815 (NI810, NI811, NI812 for DMSO and NI813, NI814, and NI815 for NOCO) using a custom shell script. Reads mapping to ribosomal RNAs were removed by mapping demultiplexed samples to rRNA reference sequences downloaded from Saccharomyces Genome Database (SGD) using the following bowtie2 (v 2.3.5.1) command: “bowtie2 -q—local -N 1 -L 15 -p 12”. PCR duplicates were then removed from the unaligned reads using fastx_collapser (v. 0.0.13). Deduplicated reads were mapped back to S288C reference genome (“S288C_reference_genome_R64-3-1_20210421”) downloaded from SGD. featureCounts (v. 2.0.1) was used to quantify number of reads mapping to genes using the annotation file “saccharomyces_cerevisiae_R64-3-1_20210421.gff” downloaded from SGD and the following command: “featureCounts -t gene -g ID -s 1”. Count tables were then imported into R (v. 4.0.3) for downstream analysis. DESeq2 (v. 1.30.1) was used to quantify differential total mRNA expression between DMSO and nocodazole conditions and riborex v.2.3.4 (Li et al., 2017) was used to quantify translational regulation between DMSO and nocodazole conditions. A single nocodazole treated total mRNA library was excluded from downstream analysis due to low sequencing depth.

### Immunoblotting

Strain SBY20970 was cultured in YEP +1% adenine + 2% glucose at 23°C to an OD_600_ of ∼0.4, at which point the culture was split into auxin + DMSO or auxin + nocodazole for 2.5 h to an OD600 of ∼0.8. 1 ml of culture from each condition was harvested and yeast were pelleted before being lysed by bead beating pellets in SDS sample buffer. Samples were then separated by SDS-PAGE. For immunoblotting, proteins were then transferred from SDS-PAGE gels onto 0.2 uM nitrocellulose at 4°C for 2 h. Membranes were blocked at room temperature with 5% milk in PBST and incubated overnight in primary antibody at 4°C. The following antibodies were used: Anti-GFP (Living Colors GFP monoclonal antibody; 1:20,000) and anti-PGK1 (Thermofisher clone 22C5D8 monoclonal antibody; 1:10,000). After overnight incubation in primary antibody, membranes were washed 2× with PBST for 15 min and then incubated with secondary antibodies (anti-mouse Igg peroxidase-linked whole secondary antibody, Cytiva; 1:10,000 dilution) for 1 h at room temperature in 5% milk in PBST. Membranes were washed 3× for 5 min in PBST and imaged.

## Results

### Microtubule disruption leads to translational and transcriptional repression of a subset of genes

**FIGURE 1:**
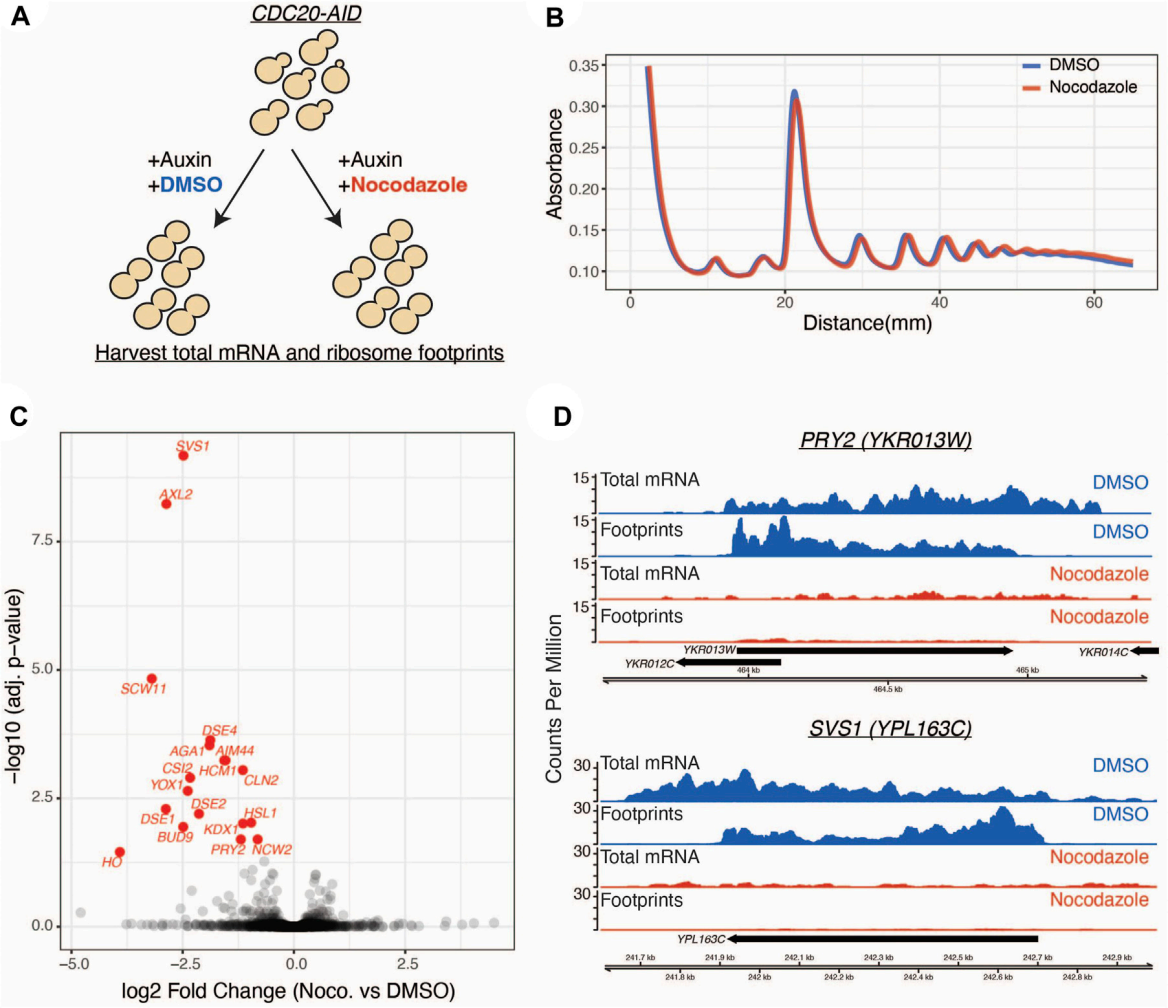
Ribosome profiling reveals a nocodazole induced translational response. **(A)** *CDC20-AID* cells (SBY14004) were split into two conditions and simultaneously treated with auxin to arrest cells in metaphase and either nocodazole or DMSO. Lysates from these populations were used for ribosome profiling and mRNA sequencing. **(B)** Polysome profiles generated from DMSO (blue) and nocodazole (red) treated cells. y-axis is absorbance at 254 nm and x-axis is millimeters (mm) from top of gradient. **(C)** Volcano plot of ribosome profiling data. Log2 fold changes of total mRNA-normalized footprint abundance (nocodazole vs. DMSO) are plotted on the x-axis, and negative log10 multiple-test corrected *p*-values are plotted on the y-axis. Individual genes are represented as black dots, and significantly translationally regulated genes (Wald test, Benjamini Hochberg adjusted *p* < 0.05) are colored in red. **(D)** Genome browser tracks of ribosome profiling and mRNA sequencing data for two representative genes. Data from the DMSO control condition is shown in blue and nocodazole condition in shown in red. Aggregate (across all replicates) counts per million are plotted on the y-axis. Genomic coordinates are plotted along the x-axis.

To determine if there is translational regulation of mitotic pathways upon microtubule disruption, we performed ribosome profiling on a population of mitotically arrested yeast cells treated with and without the microtubule destabilizing drug nocodazole (Figure 1A). To arrest cells in mitosis, we used strains where the APC activator Cdc20 was fused to an auxin-inducible degron at its endogenous locus (*CDC20-AID*) (Miller et al., 2016). We simultaneously shifted asynchronous cells into auxin in the presence or absence of nocodazole and then performed ribosome profiling, which uses deep sequencing of ribosome-protected RNA fragments to estimate ribosome occupancy and infer translation of an mRNA (Ingolia et al., 2009). We first determined if nocodazole treatment has a global inhibitory effect on translation, as this has been previously reported to occur in mammalian cells (Prescott and Bender, 1962; Fan and Penman, 1970; Stumpf et al., 2013; Tanenbaum et al., 2015). The polysome profiles of mitotically arrested cells were similar in the presence or absence of nocodazole (Figure 1B), consistent with prior studies of microtubule disruption in yeast that did not detect a global inhibition of translation upon microtubule disruption (Sweet et al., 2007).

**FIGURE 2:**
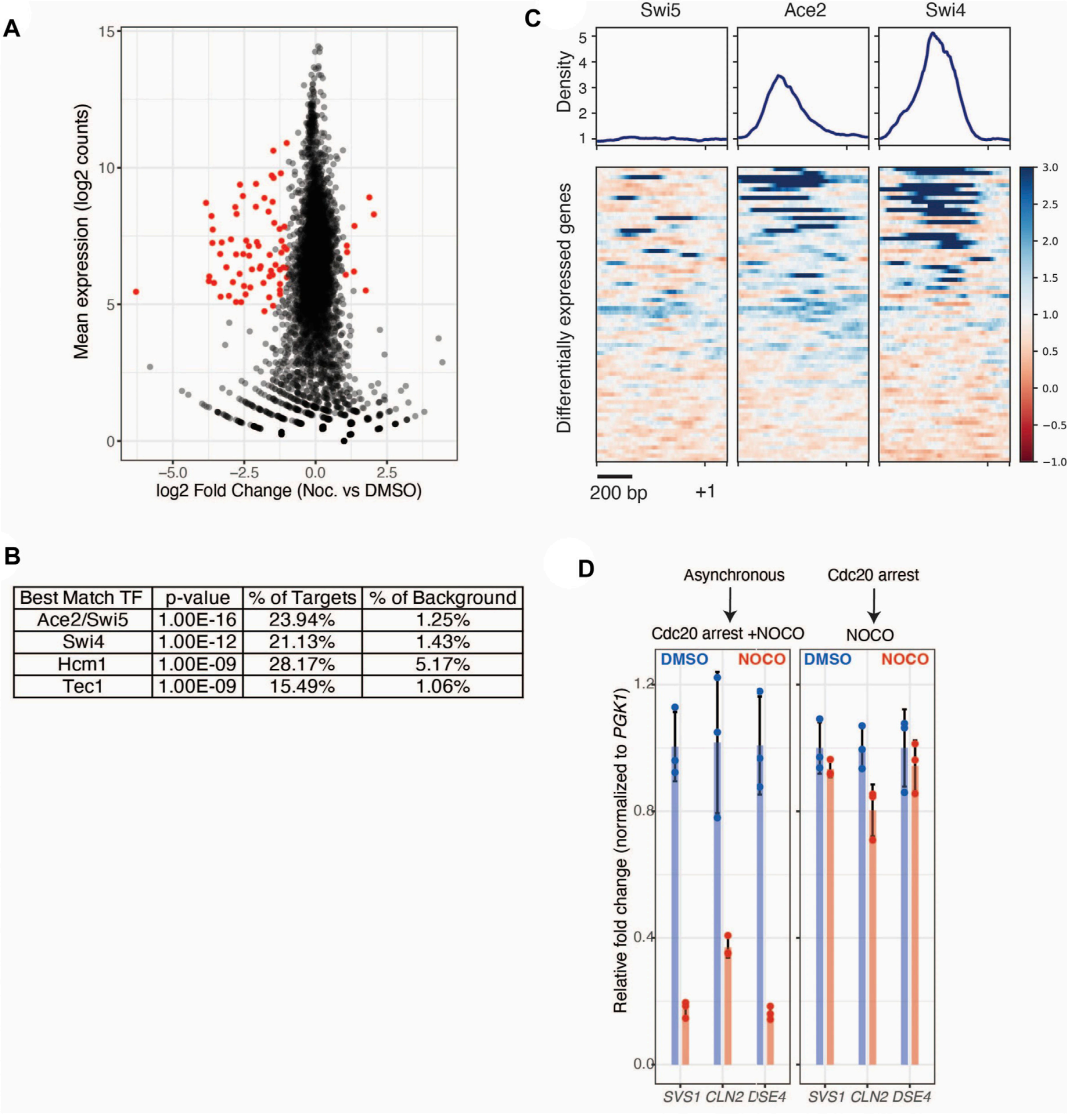
Nocodazole treatment induces transcriptional regulation of Ace2 target genes. **(A)** mRNA-seq data from *cdc20-AID* (SBY14004) cells treated with DMSO or nocodazole. Altered expression of genes upon nocodazole treatment. Log2 mean counts across all replicates and conditions plotted on the y-axis, with log2 fold change (nocodazole vs. DMSO) plotted on the y-axis. Individual genes are represented as black dots, and differentially expressed genes (Wald test, Benjamini Hochberg adjusted *p* < 0.05 and fold-change > 2) are colored in red. **(B)** Motif discovery analysis (HOMER) identification of the top 5 significant transcription factor motifs enriched within promoters of differentially expressed genes. **(C)** Binding profile and heatmap of Swi5, Ace2, and Swi4 ChIP-seq (Rossi et al., 2021) across the promoters of differentially expressed genes from **(A)**. Each row represents a 1.2 kb window (−1 kb to +0.2 kb) around a given gene’s start codon and heatmap is sorted by maximum ChIP signal for each promoter. **(D)** Nocodazole induced decrease in gene expression in *CDC20-AID* cells (SBY14004) treated with auxin and either nocodazole (10 μg/ml; Red) or DMSO (Blue). Cells were simultaneously treated with auxin and drug (left) or were sequentially treated (right). N = 3 independent replicates for each treatment. qRT-PCR quantification of individual gene expression values (*SVS1, CLN2, DSE4*) were normalized to *PGK1* expression values within the same sample and fold change is relative to DMSO controls. Error bars represent standard deviation.

Having observed no broad changes to polysome profiles in cells treated with nocodazole, we next looked for gene-level changes to ribosome occupancy. We identified 18 genes that are negatively regulated at the translational level in response to nocodazole (FDR < 0.05; Figure 1C; Supplementary Figure S1A). In control conditions, these genes were translated at appreciable levels (two representative genes are shown in Figure 1D). However, upon nocodazole treatment, ribosome footprint abundance along these transcripts was dramatically reduced, suggesting that microtubule disruption negatively impacts protein production for these specific genes (Figure 1D). In addition to altered levels of ribosome density along the affected transcripts, we also observed a stark decrease in total mRNA abundance for these genes (Figure 1D). This led us to investigate changes in global gene expression upon nocodazole treatment in the mitotically arrested cells. By analyzing the input mRNA-sequencing libraries generated for ribosome profiling, we identified 71 differentially expressed genes (FDR < 0.05, fold change > 2) (Figure 2A; Supplementary Figure S2A). Of the 18 differentially translated genes we identified *via* ribosome profiling, 14 are also significantly downregulated at the transcriptional level.

### Microtubule disruption alters regulation of Ace2 morphogenesis pathway gene expression

To determine if the affected genes are associated with a certain biological process, we performed Gene Ontology analysis on the translationally and transcriptionally regulated genes (Supplementary Figures 1B, 2B, respectively). Both analyses showed that genes involved in cell wall biology are significantly enriched. To identify transcription factors potentially responsible for the observed changes in gene expression, we performed motif discovery analysis [HOMER; (Heinz et al., 2010)] on the promoters of the differentially expressed genes. Compared to the promoters of all genes, the promoters of the differentially expressed genes are significantly enriched for binding motifs for the RAM pathway transcription factor Ace2 as well as the Swi5, Swi4, Hcm1, and Tec1 transcription factors (Figure 2B). To orthogonally validate this observation, we asked if these transcription factors are known to bind to the promoters of the differentially expressed genes using publicly available ChIP-seq data for Swi5, Ace2, and Swi4 (Rossi et al., 2021). We found that Ace2 and Swi4 bind to a subset these promoters, consistent with their binding motifs being enriched in our dataset (Figure 2C). However, Swi5 did not appear to bind to the promoters of the differentially expressed genes, consistent with prior work demonstrating that despite being paralogs with similar binding motifs, Swi5 and Ace2 target distinct sets of genes (Dohrmann et al., 1992; Doolin et al., 2001).

The enrichment of the Ace2 transcription factor motif within the promoters of the nocodazole-induced differentially expressed genes, coupled with the observation that genes involved in cell wall biology are enriched in our ribosome profiling and mRNA-seq datasets, led us to hypothesize that nocodazole could be affecting the RAM pathway (Racki et al., 2000; Bidlingmaier et al., 2001; Colman-Lerner et al., 2001; Weiss et al., 2002). However, the RAM pathway is not known to be active during metaphase, which is the stage where we harvested cells for ribosome profiling and mRNA sequencing. Because we performed these experiments by simultaneously shifting an asynchronously growing population of cells into auxin and nocodazole until they arrested two and a half hours later, we considered the possibility that the nocodazole-induced effects we detected occurred at a different cell cycle stage prior to the final metaphase arrest. To test this, we performed a quantitative real-time PCR assay (qRT-PCR) to analyze total mRNA levels on three candidate genes (*SVS1, CLN2, DSE4*) that we detected in our genomic analyses. We compared the initial experimental condition, where asynchronous cells were shifted into auxin and nocodazole simultaneously, to a condition where cells were first arrested in metaphase prior to treating them with nocodazole. Using a qRT-PCR assay, we confirmed the nocodazole-induced decreased expression of the three candidate genes when asynchronous cells were simultaneously shifted into auxin and nocodazole (Figure 2D). In contrast, there was little change in gene expression when nocodazole was added to cells that had been arrested in mitosis first. This suggests that our initial protocol reported on regulation at a cell cycle phase other than metaphase, consistent with the possibility that nocodazole affects the RAM pathway that works upon mitotic exit.

### Cbk1 localization is altered by microtubule disruption

**FIGURE 3:**
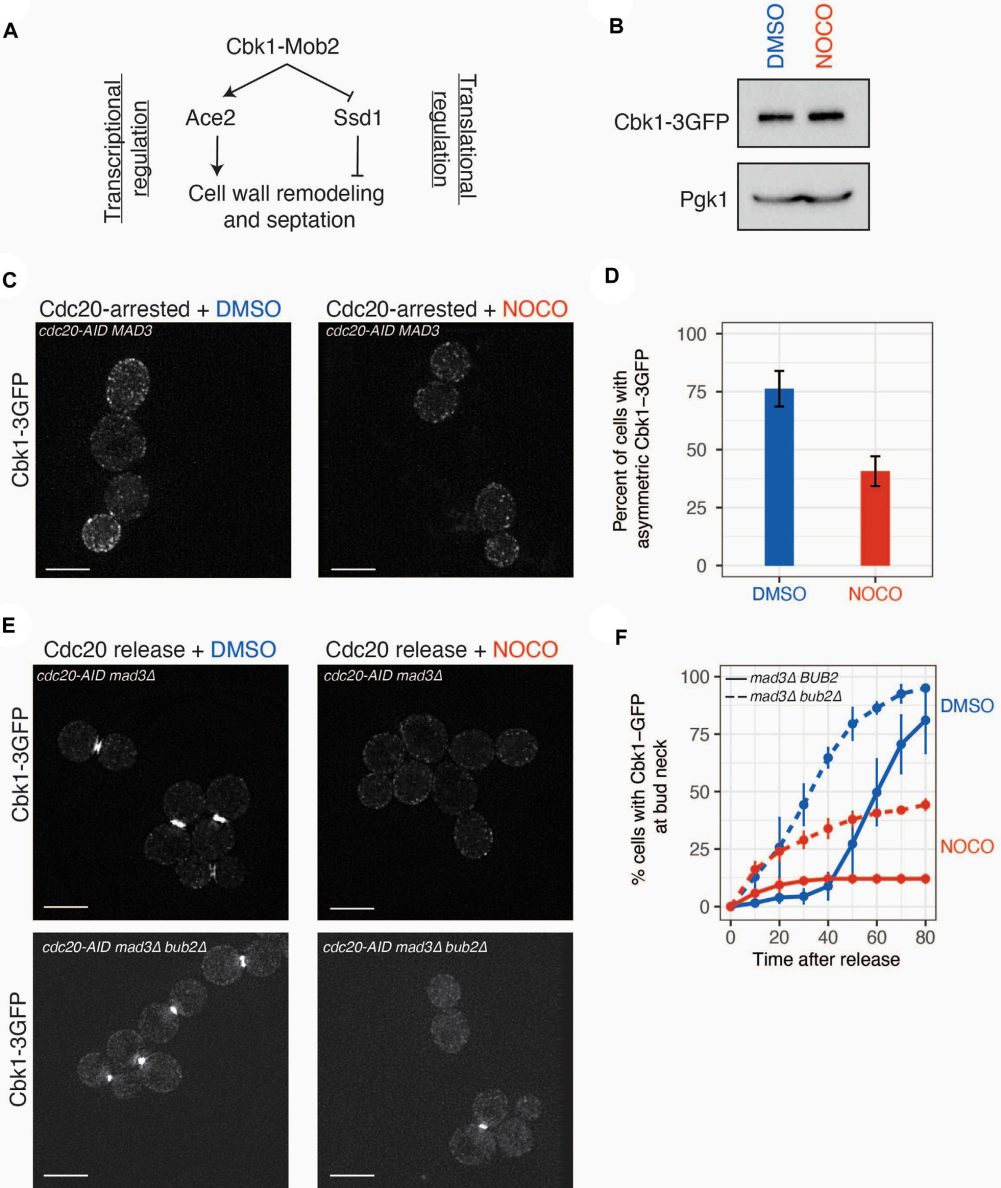
Cbk1 localization is altered in nocodazole treated cells. **(A)** Model of Cbk1 signaling pathway controlling transcription and translation of genes involved in cell wall remodeling. **(B)** Immunoblot with anti-GFP antibodies from whole cell extracts of *CDC20-AID* arrested cells (SBY20970) treated with DMSO or nocodazole for 2.5 h to analyze Cbk1-GFP levels. Pgk1 is a loading control. **(C)** Nocodazole diminishes accumulation of Cbk1-3GFP in daughter cells. *CDC20-AID* cells (SBY20970) were arrested in mitosis and treated with DMSO or nocodazole (10 μg/ml) for 2.5 h and live imaged on agarose pads. Scale bar, 5 μm. Two representative images. **(D)** Binning of Cbk1-3GFP localization phenotype. N = 3 independent replicates, error bars represent standard deviation. *p*-value derived from two-tailed Student’s *t*-test. **(E)** Nocodazole prevents accumulation of Cbk1-3GFP at the bud neck in late mitosis. *CDC20-AID mad3∆* (SBY21032) or *CDC2020-AID mad3∆ bub2∆* (SBY21080) strains were arrested in mitosis (auxin) for 2.5 h and then released into media containing DMSO or nocodazole and live-imaged on agarose pads containing DMSO or nocodazole. Scale bar, 5 μm. Two representative images 80 min post-release. **(F)** Cumulative accumulation of Cbk1-3GFP at bud neck within the population. N = 3 independent replicates, error bars represent standard deviation. Blue = DMSO, Red = nocodazole. *p*-values derived from two-tailed Student’s *t*-test.

The RAM pathway exerts transcriptional and translational control *via* the Cbk1 kinase that both activates Ace2-mediated transcription and relieves Ssd1 translational inhibition (Figure 3A). Consistent with this, 9 of the 18 translationally altered mRNAs in our dataset were previously demonstrated to be Ssd1 targets (Hose et al., 2020). However, the yeast strain background we used for our experiments is hypomorphic for some Ssd1 functions, making it unclear whether the effect of nocodazole could be mediated by Ssd1 in this strain (Sutton et al., 1991; Kaeberlein and Guarente, 2002; Mir et al., 2009). We therefore tested whether the nocodazole-induced effects on gene expression occurred in a strain background with fully functional Ssd1 (Sutton et al., 1991). There was similar inhibition of gene expression of three candidate genes in both strains, supporting the possibility that RAM signaling can work through the hypomorphic Ssd1 allele in the strain background where we initially performed the ribosome profiling (Supplementary Figure S2C).

To determine whether microtubule disruption affects the RAM pathway, we focused on Cbk1 because it regulates both Ace2 and Ssd1. First, we examined total Cbk1 protein levels in mitotically arrested cells with and without nocodazole and found they were the same in both conditions, indicating that any defect in Cbk1 signaling is not due to altered Cbk1 levels (Figure 3B). We next tested whether Cbk1 localization is perturbed upon nocodazole treatment. As mitosis progresses, Cbk1 accumulates in the daughter cell and then re-localizes to the daughter cell nucleus and the bud neck after anaphase (Colman-Lerner et al., 2001; Weiss et al., 2002). The enrichment of Cbk1-GFP in daughter cells was reduced upon nocodazole treatment of metaphase-arrested cells, consistent with altered Cbk1 activity (Figures 3C,D; *p* = 0.003808, Student’s *t*-test). To analyze the bud neck localization of Cbk1 after chromosome segregation, we released cells from a *cdc20-AID* arrest into the presence or absence of nocodazole. These cells were also deleted for the *MAD3* spindle checkpoint gene to allow cells to proceed into anaphase in the presence of nocodazole. In control cells, we observed robust Cbk1-3GFP localization to the bud neck in the majority (>85%) of cells within 80 min after release. However, in nocodazole-treated cells, this localization was essentially lost (<15% of cells) (Figures 3E,F). Previous work showed that the Cbk1 interacting protein Mob2 localizes to the bud neck after mitotic exit (Weiss et al., 2002). Because microtubule disruption after metaphase inhibits mitotic exit, we considered the possibility that Cbk1 bud neck localization also requires progression through mitosis. To test this, we deleted *BUB2* to allow activation of the mitotic exit network in the presence of nocodazole (Bloecher et al., 2000; Pereira et al., 2000; Wang et al., 2000). Deletion of *BUB2* was able to partially rescue the Cbk1-3GFP bud neck localization pattern seen in nocodazole-treated cells (Figure 3F; *p* = 0.0009921, Student’s *t*-test), suggesting that mitotic progression allows partial Cbk1 localization to the bud neck. However, it was not fully restored, suggesting that additional mechanisms that are altered by defects in the microtubule cytoskeleton regulate the localization of Cbk1 to the bud neck.

### Gene expression in the regulation of Ace2 morphogenesis pathway is altered by microtubule depolymerization

**FIGURE 4:**
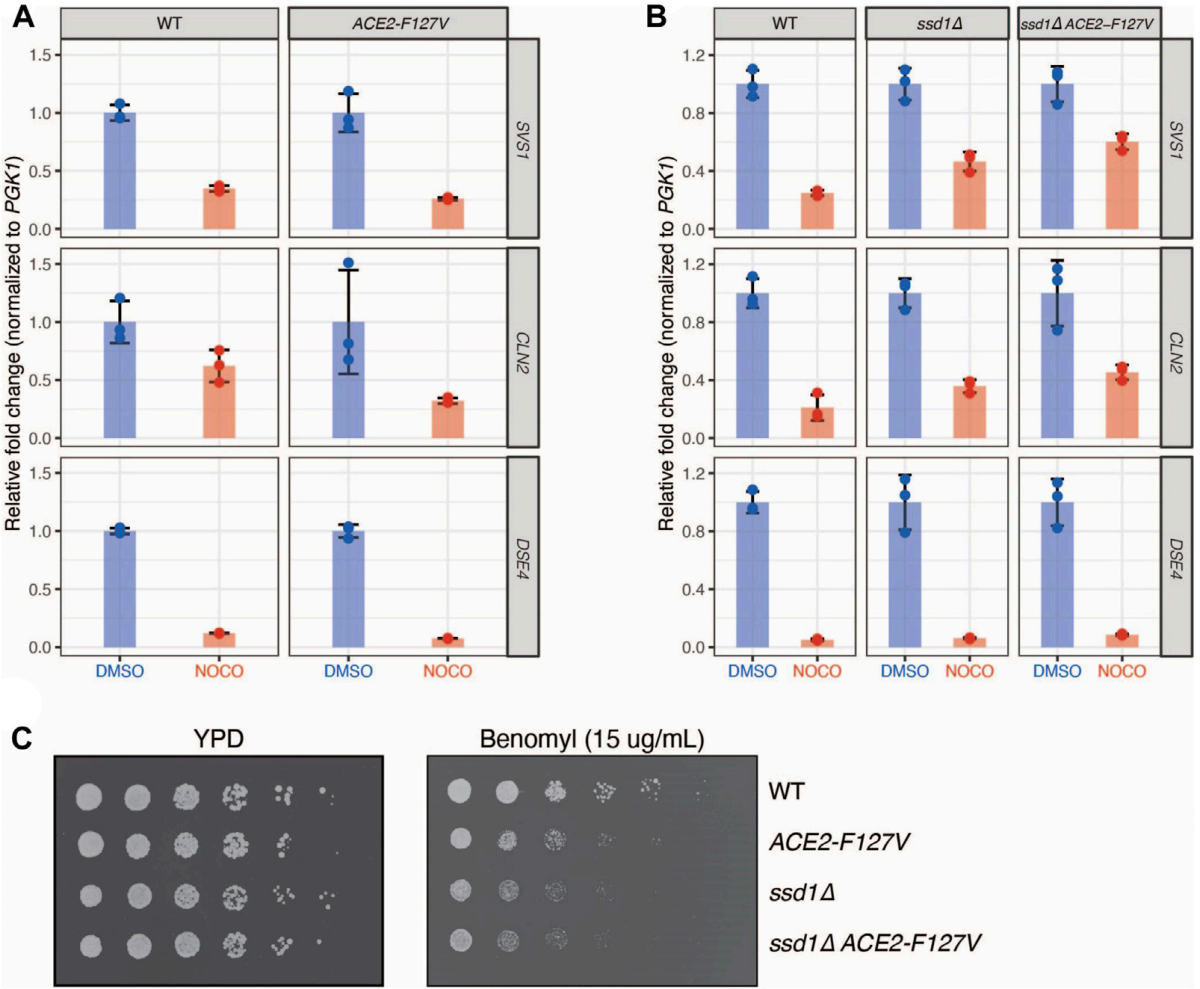
Nocodazole induced regulation of gene expression is mediated by Cbk1 signaling pathway. **(A)** A dominant allele of *ACE2* is not sufficient to rescue nocodazole induced changes in gene expression. WT (SBY21046) and *ACE2-F127V* (SBY21047) cells were treated with DMSO (Blue) or nocodazole (10 μg/ml; Red). N = 3 independent replicates for each treatment. qRT-PCR quantification of individual gene expression values (*SVS1, CLN2, DSE4*) were normalized to *PGK1* expression values within the same sample and fold change is relative to genotype-matched DMSO controls. Error bars represent standard deviation. Expression levels are not elevated in *ACE2-F127V* strains compared to WT upon nocodazole treatment (*p* = 0.99, 0.97, 0.99 for *SVS1, CLN2,* and *DSE4* respectively; two-sample *t*-test). **(B)** Deletion of *SSD1* partially rescues nocodazole-induced changes in gene expression. WT (SBY21118), *ssd1∆* (SBY21138), and *ssd1∆ ACE2-F127V* (SBY21137) were treated with DMSO (Blue) or nocodazole (10 μg/ml; Red). N = 3 independent replicates for each treatment. qRT-PCR quantification of individual gene expression values (*SVS1, CLN2, DSE4*) were normalized to *PGK1* expression values within the same sample and fold change is relative to genotype-matched DMSO controls. Error bars represent standard deviation. Expression levels are elevated in strains compared to WT upon nocodazole treatment (*p* < 0.05 for *SVS1, CLN2,* and *DSE4* in both mutant backgrounds; two-sample *t*-test). **(C)** Modulation of downstream Cbk1 targets sensitizes cells to the microtubule destabilizing drug benomyl. 5-fold serial dilutions of WT (SBY21118), *ACE2-F127V* (SBY21119), *ssd1∆* (SBY21138), and *ssd1∆ ACE2-F127V* (SBY21137) were plated onto YPD or YPD benomyl (15 μg/mL) plates and incubated at 23°C for 3 days.

To further explore whether the microtubule-based effects on gene expression are due to altered RAM pathway activity, we considered the two known Cbk1 activities that could be inhibited to alter gene expression, which are 1) activation of the Ace2 transcription factor and 2) inhibition of the Ssd1 translational inhibitor, which also destabilizes mRNA (Jansen et al., 2009). To test whether these activities are required to repress the genes affected by microtubule disruption, we used two pathway mutants: 1) an *ACE2-F127V* dominant mutant that bypasses the need for Cbk1 regulation and constitutively promotes downstream target gene transcription and 2) an *ssd1∆* mutant that promotes translation of target genes even in the presence of Cbk1 inhibition (Racki et al., 2000; Mazanka et al., 2008; Jansen et al., 2009). The dominant *ACE2-F127V* mutant was not sufficient to rescue total mRNA levels of *SVS1, CLN2,* or *DSE4* upon nocodazole treatment (Figure 4A). We considered that translational repression might be epistatic to transcriptional activity induced through *ACE2-F127V* because changes in translation can lead to transcript degradation which might be the underlying reason for decreased transcription (Deana and Belasco, 2005; Edri and Tuller, 2014). To test this, we deleted *SSD1* and found it was indeed able to partially rescue total mRNA levels of *SVS1* and *CLN2*, and when combined with *ACE2-F127V*, this rescue was synergistic (Figure 4B). These results are consistent with the repression being regulated at both the transcriptional and translational levels by the RAM pathway. However, despite the rescue of *SVS1* and *CLN2* expression, we found no alteration of *DSE4* levels in the mutant backgrounds (Figure 4B). Together, these data indicate that a subset of genes affected by nocodazole may be the result of altered Cbk1 signaling and that additional unidentified mechanisms also exist.

Our finding that microtubule disruption inhibits the expression of genes involved in cell wall biology suggested that this might be important to prevent premature cell separation and G1 entry. If this were true, constitutive expression of these genes in the presence of nocodazole should be deleterious to cells. To test this, we plated serial dilutions of WT, *ACE2-F127V, ssd1∆*, and *ssd1∆ ACE2-F127V* cells onto the microtubule drug benomyl and found that dominant activation of this pathway indeed led to benomyl sensitivity (Figure 4C). We attempted to determine if septation timing is altered in this situation by monitoring zymolyase sensitivity but did not obtain reproducible results. Although the underlying mechanism is not yet known, our data identify a role for microtubule integrity in regulating the RAM pathway through signaling pathways that help to ensure the coordination of mitotic events.

## Discussion

### Regulation of transcription and translation in response to microtubule disruption

Here we report the first global analysis of budding yeast translation in the presence and absence of microtubule disruption. Because we induced a mitotic arrest at the same time we added the microtubule depolymerizing agent, we fortuitously identified a class of genes that are regulated by microtubule integrity after mitotic exit. Supporting this idea, when we arrested cells in metaphase prior to adding the microtubule destabilizer, we did not detect this regulation. The class of genes that are downregulated both transcriptionally and translationally in the absence of microtubules are enriched in cell wall processes. We envision at least two non-mutually exclusive ways in which this coordinated downregulation could take place. First, loss of ribosome density itself could lead to a secondary effect of increased transcript degradation and decay, thus leading to an observed decrease in signal in both ribosome profiling and mRNA sequencing experiments. This phenomenon has been observed in both prokaryotes and eukaryotes (Deana and Belasco, 2005; Edri and Tuller, 2014). A second mechanism that could mediate a coordinated downregulation of transcription and translation of a message is a feed-forward signaling pathway that acts at both levels of gene expression. One such signaling pathway is the RAM pathway that regulates the transcription and translation of genes involved in cell wall remodeling (Racki et al., 2000; Bidlingmaier et al., 2001; Colman-Lerner et al., 2001; Weiss et al., 2002; Jansen et al., 2009). This pathway is controlled by Cbk1 which regulates transcription *via* Ace2 and translation *via* Ssd1. Our data support a model in which microtubule disruption mechanistically impedes Cbk1 function, leading to downregulation of transcription of cell wall targets through Ace2 and simultaneous downregulation of translation (and likely mRNA decay/degradation) of these same targets *via* Ssd1. We hypothesize that this regulatory mechanism exists to prevent premature septation prior to completing mitosis. In support of such a hypothesis is our observation that constitutive activation of this gene expression program becomes detrimental to growth in the presence of microtubule disruption.

The Ssd1 translational inhibitor is highly polymorphic across wild and lab yeast strains (Sutton et al., 1991). In the W303 background wherein our genomic experiments were conducted, *SSD1* is prematurely truncated (*ssd1-d2*), leaving Ssd1 without its functionally annotated RNA binding domain (Uesono et al., 1997). This truncation is widely thought to render Ssd1 non-functional, as *ssd1-d2* phenocopies *ssd1∆* for some functions (Kaeberlein and Guarente, 2002). However, some *ssd1∆* phenotypes, such as zymolyase sensitivity, cellular aggregation, and thermosensitivity, are fully complemented by *ssd1-d2* (Mir et al., 2009). In addition, recent structural work demonstrated that Ssd1 binds RNA along the surface of its N-terminus in a region still intact in *ssd1-d2* (Bayne et al., 2022). These findings suggest *ssd1-d2* is likely at least partially functional. Consistent with this, we detected nocodazole-dependent repression of candidate genes in the S288C background where Ssd1 is wildtype. These results suggest that nocodazole negatively regulates the Cbk1-Ace2-Ssd1 feed forward loop that positively regulates expression of cell wall associated genes.

Although we found evidence for regulation of the Cbk1-Ace2-Ssd1 feed-forward loop by microtubule disruption, additional mechanisms must exist because some genes were not regulated by this pathway, such as *DSE4*. Consistent with this observation, Ssd1 binds to *SVS1* and *CLN2* transcripts but not to *DSE4* transcripts (Hose et al., 2020). We did identify other transcription factor motifs (e.g., for Swi4, Hcm1) enriched in the promoters of the differentially expressed genes, so it is possible that these transcription factors are also regulated by microtubule disruption. In support of this, it has previously been observed that deletion of *SWI4* protects cells against microtubule disruption, indicating that Swi4 and its downstream transcriptional targets may be deleterious in the presence of this stress (Beilharz et al., 2017). Additional translational regulators may also be acting in response to microtubule disruption in parallel to Ssd1. In a survey of RNA binding proteins, several other regulators (e.g., Scp160, Khd1/Hek2, Pub1, Nab6 and Mrn1) were found to have significantly correlated binding profiles with Ssd1 (Hogan et al., 2008). It is possible that one or more of these factors may be acting redundantly with Ssd1 to promote regulation of cell wall encoding messages. Finally, another possible mechanism of regulation could be through P-bodies, cytoplasmic sites of post-transcriptional and translational gene regulation. It was previously observed that chemical or genetic perturbation of microtubule function in yeast led to the formation of P-bodies (Sweet et al., 2007). Understanding if the induction of P-bodies upon microtubule stress, as well as the RNA and protein contents of these P-bodies, is linked to the gene regulation uncovered in this study will help further elucidate the mechanism by which cells respond to microtubule stress.

### Microtubule integrity regulates Cbk1 localization and activity

Early in the cell cycle, Cbk1 localizes to sites of polarized growth and active cell wall remodeling (Colman-Lerner et al., 2001; Weiss et al., 2002). As mitosis proceeds, Cbk1 localizes to the daughter cell and then relocalizes to the daughter cell nucleus and the bud neck after mitosis (Colman-Lerner et al., 2001; Weiss et al., 2002). Our data show that Cbk1 localization to the daughter cell and the bud neck is sensitive to disruption of the microtubule cytoskeleton. We did not detect Cbk1 in the daughter nucleus in our experiments, likely due to the transient nature of this localization event. However, it is likely that the altered daughter cell localization results in a defect in Cbk1 nuclear localization, consistent with our finding that there is decreased transcription of Ace2 targets. It is unclear whether microtubules directly regulate Cbk1 localization or whether it is an indirect effect of signaling mechanisms that are activated in response to microtubule defects. Consistent with the latter possibility, Cbk1 localization to the bud neck was partially restored when nocodazole-treated cells were allowed to exit mitosis by inhibiting the spindle position checkpoint. In addition, prolonged mitotic arrest leads to altered Ace2 localization, which may also contribute to altered transcription (Herrero et al., 2020). However, mechanisms in addition to mitotic exit are likely involved in regulating Cbk1 because many nocodazole-treated cells still had defective Cbk1 localization despite activation of the mitotic exit network. One possibility is that the bud neck localization of Cbk1 requires nuclear division, a process that cannot occur without microtubules even when the mitotic exit network is activated. In the future, it will be important to elucidate the underlying mechanisms whereby microtubule integrity regulates Cbk1 localization.

Spatiotemporal regulation of Cbk1 localization suggests that a mechanism of localized translational regulation, a common regulatory paradigm widely conserved across eukaryotes, is important for proper completion of mitosis. In support of such a hypothesis is our observation that constitutive activation of this gene expression program becomes detrimental in the presence of microtubule disruption. We hypothesize that this regulatory mechanism exists to prevent premature septation prior to correct completion of mitosis, which would potentially creating an anucleate daughter cell, a nonreversible and deleterious outcome. In the future, it will be important to further understand the additional regulatory mechanisms that ensure appropriate gene expression throughout mitosis as well as to further elucidate the underlying regulation of the RAM pathway by the microtubule cytoskeleton.

## Data Availability

The data presented in the study are deposited in the SRA repository, accession numbers SRR18277492 and SRR18277493.
