# Supplementary material for: Microtubule integrity regulates budding yeast RAM pathway gene expression
Source: Front Cell Dev Biol. 2022 Sep 12;10:989820. doi: 10.3389/fcell.2022.989820 (PMC9511886; doi:10.3389/fcell.2022.989820)
Supplement: Supplementary file 1 [file DataSheet1.zip › zip file supplementary/Supplementary Figures.pdf]

Supplementary Figure 1

A

	DMSO rep 2	DMSO rep 3	NOCO rep 1	NOCO rep 2	NOCO rep 3
NOCO rep 2					0.999
NOCO rep 1				0.997	0.995
DMSO rep 3			0.996	0.991	0.988
DMSO rep 2		0.999	0.995	0.992	0.99
DMSO rep 1	0.997	0.997	0.996	0.993	0.991

B

GOID	TERM	CORRECTED_PVALUE
GO:0071944	cell periphery	7.63E-07
GO:0009277	fungal-type cell wall	7.83E-07
GO:0005618	cell wall	8.30E-07
GO:0030312	external encapsulating structure	8.30E-07
GO:0005935	cellular bud neck	1.01E-06
GO:0030427	site of polarized growth	1.07E-06
GO:0005933	cellular bud	8.22E-06
GO:0005576	extracellular region	1.27E-05
GO:0000322	storage vacuole	1.14E-03
GO:0000323	lytic vacuole	1.14E-03
GO:0000324	fungal-type vacuole	1.14E-03
GO:0005773	vacuole	2.81E-03
GO:0000399	cellular bud neck septin structure	4.95E-03
GO:0032161	cleavage apparatus septin structure	4.95E-03

Supplementary Figure 1: Ribosome profiling identifies cell-wall associated transcripts as translationally regulated upon nocodazole treatment.

(**A**) Correlation matrix of ribosome footprint libraries. (**B**) GO term (Process) analysis identifies several significantly enriched terms among our translationally regulated genes.

Supplementary Figure 2

A

	DMSO rep 2	DMSO rep 3	NOCO rep 1	NOCO rep 2
DMSO rep 1	0.996	0.995	0.994	0.993
DMSO rep 2		0.998	0.988	0.991
DMSO rep 3			0.988	0.992
NOCO rep 1				0.996

B

GOID	TERM	CORRECTED_PVALUE
GO:0000786	nucleosome	6.32E-15
GO:0009277	fungal-type cell wall	6.97E-15
GO:0005618	cell wall	8.04E-15
GO:0030312	external encapsulating structure	8.04E-15
GO:0005576	extracellular region	8.77E-13
GO:0044815	DNA packaging complex	1.26E-12
GO:0005933	cellular bud	1.22E-09
GO:0031298	replication fork protection complex	2.32E-08
GO:0031225	anchored component of membrane	1.44E-07
GO:0044426	cell wall part	6.05E-07
GO:0044462	external encapsulating structure part	6.05E-07
GO:0071944	cell periphery	1.14E-06
GO:0032993	protein-DNA complex	1.51E-06
GO:0043596	nuclear replication fork	2.78E-06
GO:0030427	site of polarized growth	2.82E-06
GO:0005935	cellular bud neck	1.73E-05
GO:0005621	cellular bud scar	2.44E-05
GO:0005657	replication fork	2.63E-05
GO:0000785	chromatin	0.000303631
GO:0000307	cyclin-dependent protein kinase holoenzyme complex	0.001068822
GO:0000322	storage vacuole	0.002331448
GO:0000323	lytic vacuole	0.002331448
GO:0000324	fungal-type vacuole	0.002331448
GO:0005773	vacuole	0.002400145
GO:1902911	protein kinase complex	0.002505585
GO:0044454	nuclear chromosome part	0.00606889

C

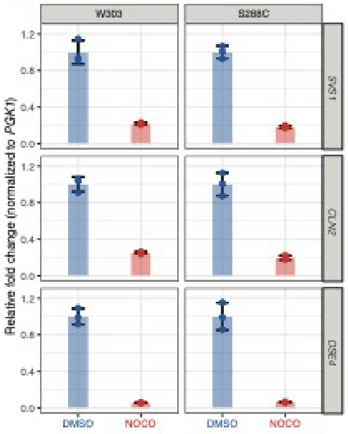

Supplementary Figure 2: mRNA-seq identifies cell-wall and cell cycle associated transcripts and negatively regulated upon nocodazole treatment.

(**A**) Correlation matrix of mRNA sequencing libraries. (**B**) GO term (Process) analysis of differentially expressed genes after nocodazole treatment. (**C**) Nocodazole induced changes to gene expression in two different genetic backgrounds. W303 (SBY3) and S288C (SBY21118) cells were treated with DMSO (Blue) or nocodazole (10ug/mL; Red). N=3 independent replicates for each treatment. qRT-PCR quantification of individual gene expression values (*SVS1, CLN2, DSE4*) were normalized to *PGK1* expression values within the same sample and fold change is relative to genotype-matched DMSO controls. Error bars represent standard deviation.
