# Supplementary material for: Microtubule integrity regulates budding yeast RAM pathway gene expression
Source: Front Cell Dev Biol. 2022 Sep 12;10:989820. doi: 10.3389/fcell.2022.989820 (PMC9511886; doi:10.3389/fcell.2022.989820)
Supplement: Supplementary file 1 [file DataSheet1.zip › zip file supplementary/Supplementary Table S1.docx]

**Supplementary Table 1.** Yeast trains used in this study.

**Strain Relevant Genotype Source**

| SBY3 (W303) | *MAT*a *ura3-1 leu2-3,112 his3-11 trp1-1 can1-100 ade2-1 bar1-1* | Biggins Lab |
| --- | --- | --- |
| SBY14004 (W303) | *MAT*a *leu2::pGPD1-OsTIR1::LEU2 CDC20-AID::KanMX* | Biggins Lab |
| SBY20970  (W303) | *MAT*a *leu2::pGPD1-OsTIR1::LEU2 CDC20-AID::KanMX CBK1-3GFP::HIS3* | This work |
| SBY21032  (W303) | *MAT*a *leu2::pGPD1-OsTIR1::LEU2 CDC20-AID::KanMX CBK1-3GFP::HIS*3 *mad3∆::HIS3* | This work |
| SBY21046  (S288C) | *MAT*a *leu2-3,11 trp1∆1 ura3-52 his3∆200* | Mazanka et al., 2008 |
| SBY21047  (S288C) | *MATɑ leu2-3,11 trp1∆1 ura3-52 his3∆200 ACE2-F127V-GFP::KANMX* | Mazanka et al., 2008 |
| SBY21080  (S288C) | *MAT*a *leu2::pGPD1-OsTIR1::LEU2 CDC20-AID::KanMX CBK1-3GFP::HIS*3 *mad3∆HIS3 bub2∆::URA3* | This work |
| SBY21118  (S288C) | *MAT*a *leu2-3,11 trp1∆1 ura3-52 his3∆200* | This work |
| SBY21119  (S288C) | *MAT*a *ACE2-F127V-GFP::KANMX* | This work |
| SBY21137  (S288C) | *MAT*a *ACE2-F127V-GFP::KANMX ssd1∆HIS3* | This work |
| SBY21138  (S288C) | *MAT*a *ssd1∆HIS3* | This work |
